# Three-body aggregation of guest molecules as a key step in methane hydrate nucleation and growth

**DOI:** 10.1038/s42004-022-00652-0

**Published:** 2022-03-14

**Authors:** Wenfeng Hu, Cong Chen, Jingyue Sun, Ning Zhang, Jiafei Zhao, Yu Liu, Zheng Ling, Weizhong Li, Weiguo Liu, Yongchen Song

**Affiliations:** 1 School of Energy and Power Engineering, Dalian University of Technology, 116024 Dalian, P. R. China; 2 Key Laboratory of Ocean Energy Utilization and Energy Conservation of Ministry of Education, 116024 Dalian, P. R. China; 3 School of Petroleum and Chemical Engineering, Dalian University of Technology, 124221 Panjin, P. R. China

**Keywords:** Reaction kinetics and dynamics, Energy, Molecular dynamics

## Abstract

Gas hydrates have an important role in environmental and astrochemistry, as well as in energy materials research. Although it is widely accepted that gas accumulation is an important and necessary process during hydrate nucleation, how guest molecules aggregate remains largely unknown. Here, we have performed molecular dynamics simulations to clarify the nucleation path of methane hydrate. We demonstrated that methane gather with a three-body aggregate pattern corresponding to the free energy minimum of three-methane hydrophobic interaction. Methane molecules fluctuate around one methane which later becomes the central gas molecule, and when several methanes move into the region within 0.8 nm of the potential central methane, they act as directional methane molecules. Two neighbor directional methanes and the potential central methane form a three-body aggregate as a regular triangle with a distance of ~6.7 Å which is well within the range of typical methane-methane distances in hydrates or in solution. We further showed that hydrate nucleation and growth is inextricably linked to three-body aggregates. By forming one, two, and three three-body aggregates, the possibility of hydrate nucleation at the aggregate increases from 3/6, 5/6 to 6/6. The results show three-body aggregation of guest molecules is a key step in gas hydrate formation.

## Abstract (web summary)

Gas hydrates have an important role in environmental and astrochemistry, as well as energy materials, but the hydrate nucleation process is not fully understood. Here, the authors use molecular dynamics simulations to show that methane molecules gather with a three-body aggregate pattern.

## Introduction

Gas hydrates have an important role in methane hydrate exploitation^1^, CO_2_ capture and separation^2^, water treatment^3^, energy storage^4^, oil and gas pipelines^5,6^, icy satellites^7^ and interstellar medium^8,9^. The mechanisms of gas hydrate nucleation and growth is essential for these applications. Understanding the pathways of gas hydrate nucleation and growth are of great scientific significance and will provide guidance for controlling strategies of hydrate formation.

Guest gas exist in the form of a large number of nanobubbles to overcome the solubility barrier when guest gas is provided in hydrate-based technologies or methane hydrate decomposition process^10–12^. The existence of nanobubbles provides a large amount of gas–water interface for hydrate nucleation and have been regarded as the origin of hydrate secondary formation during methane hydrate exploitation^13,14^. However, Although the relationship between hydrate decomposition or formation and nanobubbles has been discussed^15,16^, the influence mechanism of nanobubbles on the nucleation of hydrate from the molecular level is still open to questions.

Hypotheses for gas hydrate nucleation mechanism include: classical nucleation theories^17^, labile cluster hypothesis^18^, local structure hypothesis^19^, cage adsorption hypothesis^20^, blob hypothesis^21,22^, interface nucleation mechanism^23^, and hydration layer compression/shedding hypothesis^24^. With classical nucleation theory, the activation barrier of gas hydrate nucleation is predicted combing the free energy change due to creation of a new interface and a more stable phase^17,25^. However, the classical nucleation theory is challenged when handling excess free energy and critical radius of hydrate nucleus. And it does not provide the detailed nucleation pathway as well as the hydrate structure. The labile cluster hypothesis describes that there are labile rings of water molecules in liquid which can aid formation of the critical nucleus by agglomeration around the dissolved guest molecules^18^. Against from labile cluster hypothesis, the local structuring hypothesis describes the origin of gas hydrate nucleation as the local ordering of guest molecules caused by thermal fluctuation^19^. In cage adsorption hypothesis, gas hydrate formation is triggered by the strong attraction of water cage with guest molecules^20^. The blob hypothesis proposed that the nucleation originates from a guest rich amorphous precursor where the amorphous clathrate cage forms and dissolves until a critical nucleus forms^21^. The interface nucleation mechanism describes that guest molecules diffuse and adsorb by the gas liquid interface where a cage was generated by water molecules around the adsorbed guest molecules^23^. For the hydration layer compression/shedding hypothesis, the water hydration layers of the neighbor methane molecules are compressed to form ternary water-ring aggregations which is recognized as the fundamental structures in gas hydrate nucleation^24^. With these hypotheses, the main debate focuses on whether the gas hydrate nucleation is originally triggered by guest molecules or water molecules. There are several unresolved questions in the research of methane hydrate nucleation and growth^26^. It is well accepted that gas accumulation is an important and necessary process during hydrate nucleation and growth^19,24^. However, how the guest molecules aggregate and their aggregation patterns remains unknown.

Here, we performed a series of molecular dynamics simulations which is a powerful tool to reveal hydrate nucleation and growth pathways^24,27^. Different boxes of methane–water solutions were constructed where nanobubbles of methane gas molecules with different sizes were applied as a gas supply (Supplementary Fig. 1a). Simulation boxes were generated using randomly distributed water and methane molecules to avoid the “memory effect”^28^. The initial molar fraction of methane ranges between 0.086 and 0.148, including perfect or insufficient gas supply (Supplementary Fig. 1b). The potential energy, system configuration, hydrate growth features were analyzed using the trajectories of systems with different nanobubble sizes. The effects of nanobubble on gas hydrate nucleation and growth were then investigated. By tracking gas aggregation trajectory, the relationship between hydrate nucleation and methane aggregation patterns has been constructed. Then three-body aggregates of methane molecules were recognized to control hydrate nucleation, which were proved by inserting different numbers of three-body aggregates in a methane–water box to regulate hydrate nucleation sites and growth rates.

## Results and discussion

### Effects of nanobubble on gas hydrate nucleation and growth

**Fig. 1:**
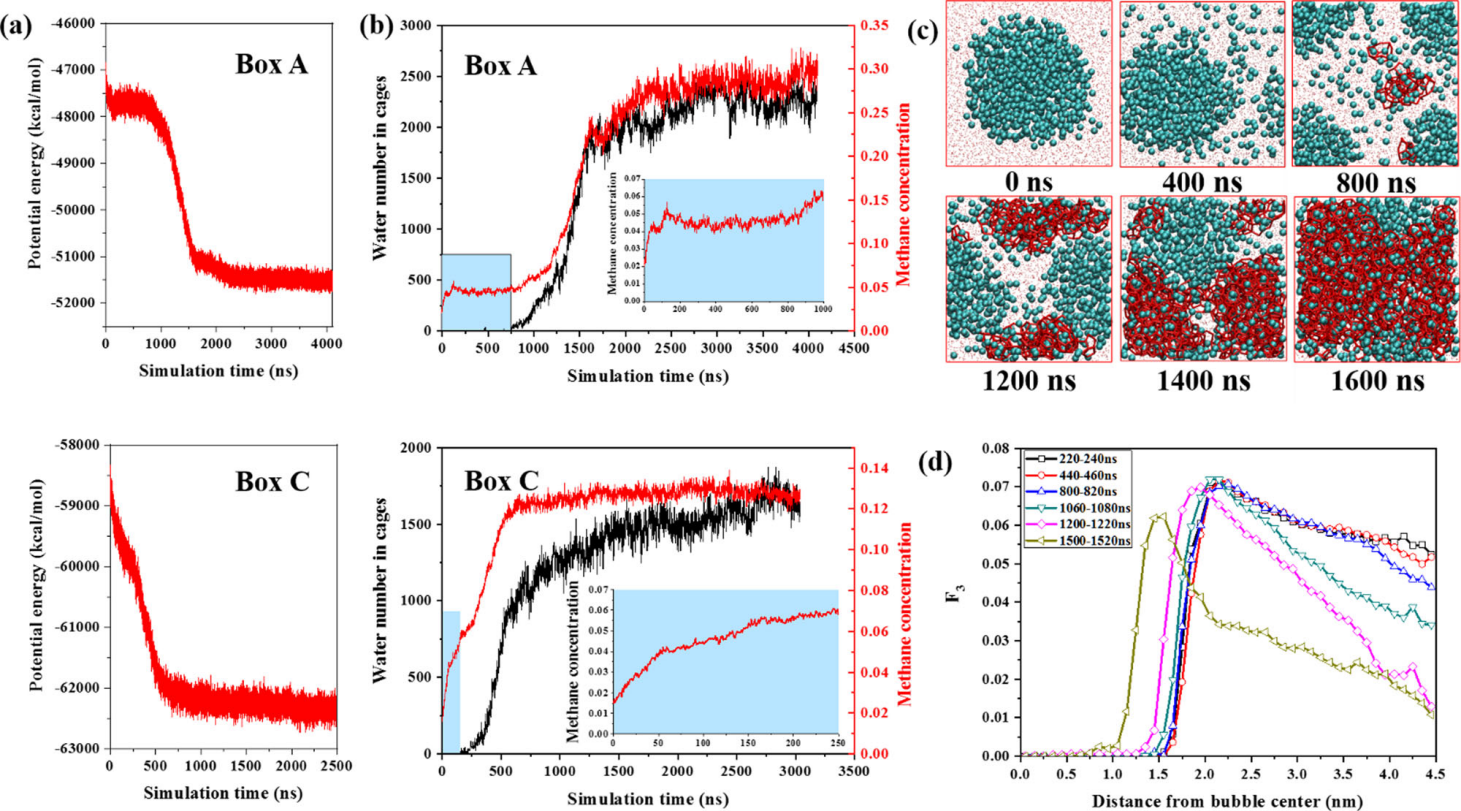
Potential energy, water number in cages, methane concentration, *F*_3_ order parameter, and system configuration along typical hydrate growth trajectories. **a** System potential energy for boxes A and C. **b** The changes of methane concentration as well as water number in cages for boxes A and C. The induction period is denoted as a marked region. The change of methane concentration in the induction period is enlarged. For box A, the concentration increases rapidly in the initial stage and then gradually maintains at a certain value, indicating that the system concentration has reached saturation, and the nucleation is triggered when the system is in a period of supersaturation. While for box C, the concentration of the system continues to increase to ~0.13 without maintaining any concentration value before nucleation starts. **c** Snapshots of a typical hydrate formation process (methane molecules are marked in cygan balls, water cages are marked in red lines). **d** The *F*_3_ order parameters in box A as a function of distance from the center of the nanobubble in different time periods.

**Fig. 2:**
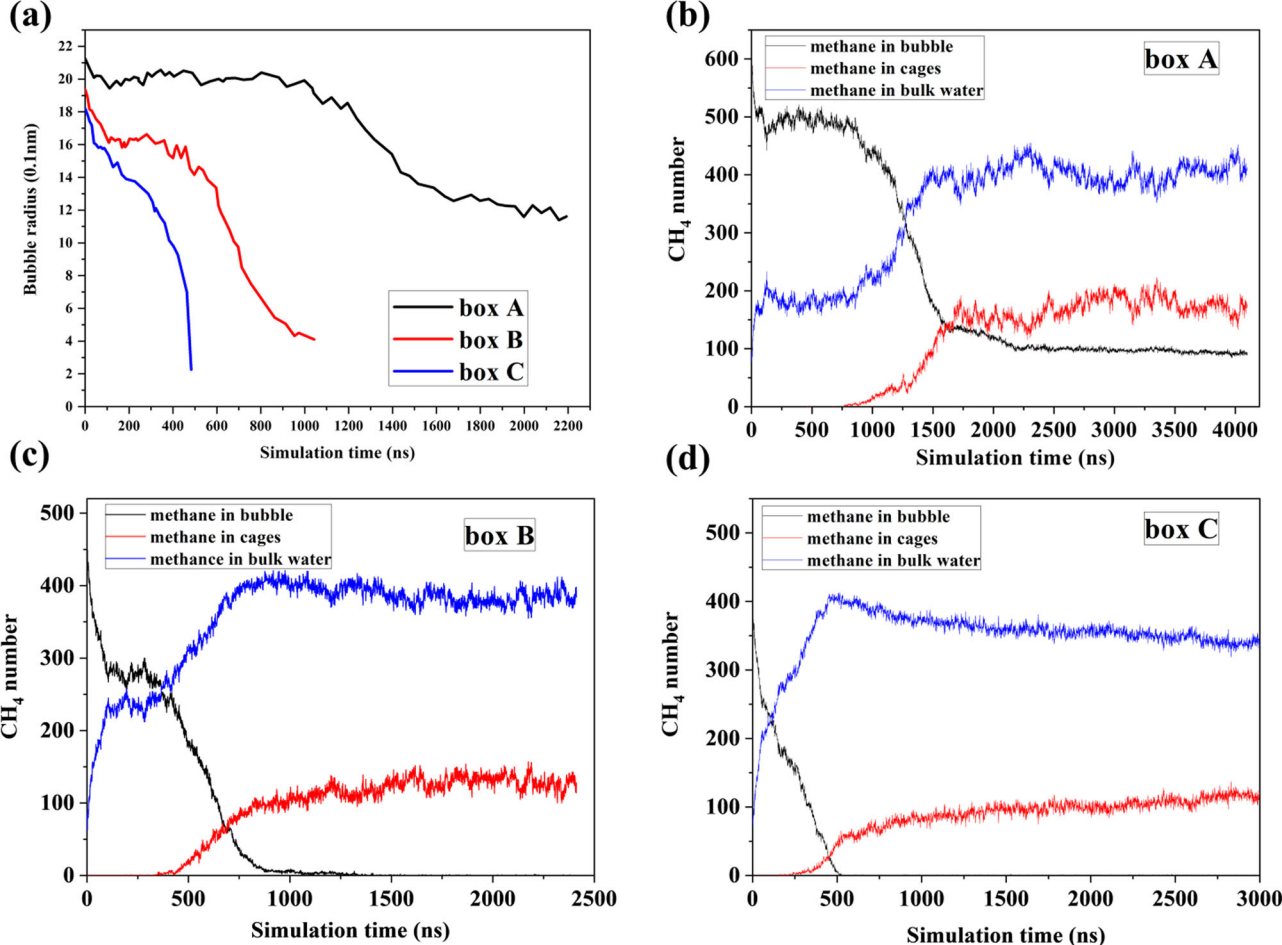
The evolution of dynamic characteristics in the process of hydrate nucleation for different boxes. **a** The changes in the bubble radius over time for boxes A–C. **b** The change in the number of methane molecules in different status for box A. **c** The change in the number of methane molecules in different status for box B. **d** The change in the number of methane molecules in different status for box C.

Figure 1a plots the potential energy along typical hydrate growth trajectories. For box A, the system potential first slightly decreases and then descends sharply after a plateau period. The decrease at the early stage is caused by methane dissolving, and the sharp descending is due to gas hydrate growth as well as accompanying methane dissolving^27^. The plateau indicates a relative equilibrium between gas bubble and water solution in the induction period. Surprisingly, the potential energy for box C descends continuously. The missing of the plateau is due to the continuously dissolving of methane as huge internal pressure makes small nanobubbles difficult to survive^29^. The different evolution behavior controlled by the initial gas–water ratio is confirmed by the variation of bubble radius (Fig. 2a) and the number of methane with different statuses (Fig. 2b–d) (refer to the “Methods” section for the identification of CH_4_ phase). The bubble still exists until hydrate no longer grows in box A, while it disappears for smaller initial gas–water ratios. Similar trends were found from six independent simulations, as illustrated in Supplementary Fig. 2. Figure 1b illustrates the changes in methane concentration as well as water number in cages. After methane dissolution, a supersaturation stage is essential for gas hydrate nucleation^28^. Although not always guarantee nucleation^28^, supersaturation is the driving force for gas hydrates crystallization^30,31^. The supersaturation concentration when hydrate nucleates for box A and C is 0.04691 and 0.05583, which are slightly larger than the data of Walsh^32^ because we use smaller bubble sizes. The supersaturation concentration proposed in this paper is also greater than critical methane concentration (0.044) which predicted by Guo et al. ^20,26^. Hydrate growth rate strongly depends on the increasing rate of methane concentration which agrees well with experiments^33^.

**Fig. 3:**
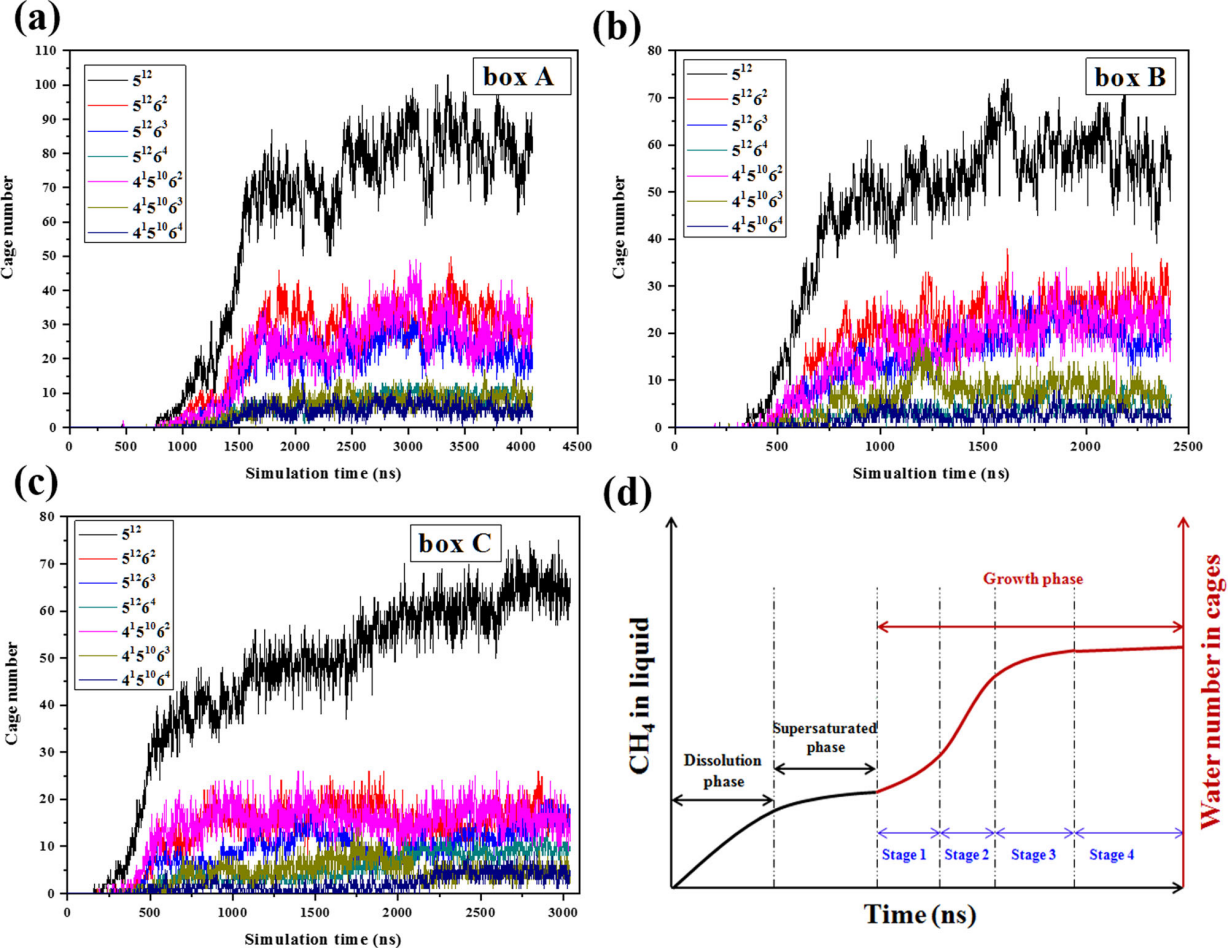
Hydrate cage number evolution with time and different hydrate growth stages. **a–c** The change of hydrate cage numbers for simulation boxes A–C. **d** An illustration of different stages for hydrate nucleation and growth. Stage 4 denotes no more hydrate grows.

Based on the evolution of water number in cages (Fig. 1b) and the cage number (Fig. 3a–c), the hydrate growth experiences three stages as shown in Fig. 3d (the schematic diagram is modified based on Khurana’s^28^): initial growth stage 1, where gas bubble collapses slowly; fast growth stage 2, where the shrinking rate of bubbles becomes faster, leading to rapid growth of hydrate; post growth stage 3, where excessive methane concentration in the bulk phase increases the resistance to continuous dissolution of bubbles and reduces the bubble collapse rate. Hydrate grows at a speed of 2–3 times faster in stage 2 compared with stage 1 (Supplementary Table 1). Figure 1c gives the typical snapshots of box A. From macroscopic experiments, hydrate grows from the gas–water interface^33^; however, it nucleates at several nm away from the interface at a molecular level (Supplementary Fig. 3a–c). A water adsorption layer has been found at flat or curved gas–water interface^32,34^. In the adsorption layer, the number density of water rings is large^34^, but the local methane concentration is relatively low (Supplementary Fig. 3d–f). As approaching the gas–water interface from 0.8 to 0.4 nm in the water side, methane concentration decreases by 61.7%, 49.1%, and 58.3% for boxes A–C, respectively. The drop of methane concentration seems to be more intense than that near flat gas–water interface^32^. Figure 1d illustrates the radial distribution of *F*_3_ order parameter in different time periods (refer to the “Methos” section for the calculation method of *F*_3_ sequence parameters). Similar to a flat interface^31^, a maximum of *F*_3_ order parameter forms at the gas–water interface. However, the *F*_3_ in the water phase gradually decreases as moving away from the interface. At 440-460 ns, the *F*_3_ at ~4.41 nm is the smallest, which agrees well with the location of the first hydrate cage, as shown in Supplementary Fig. 3a. The average *F*_3_ decreases with time and further away from the interface, it drops faster. However, before 820 ns, *F*_3_ only decreases in the zone where the radial distance from the bubble center is more than 4 nm. After 1080 ns, the interface moves towards the bubble canter as the bubble size tends to decrease (Fig. 2a). After 1200 ns, compared with those in the zone closer to the bubble center, the decreasing rate of *F*_3_ order parameter in the zone at larger distance with the bubble center becomes smaller because of hydrate growth.

### Aggregation characteristics of guest molecules during hydrate nucleation

**Fig. 4:**
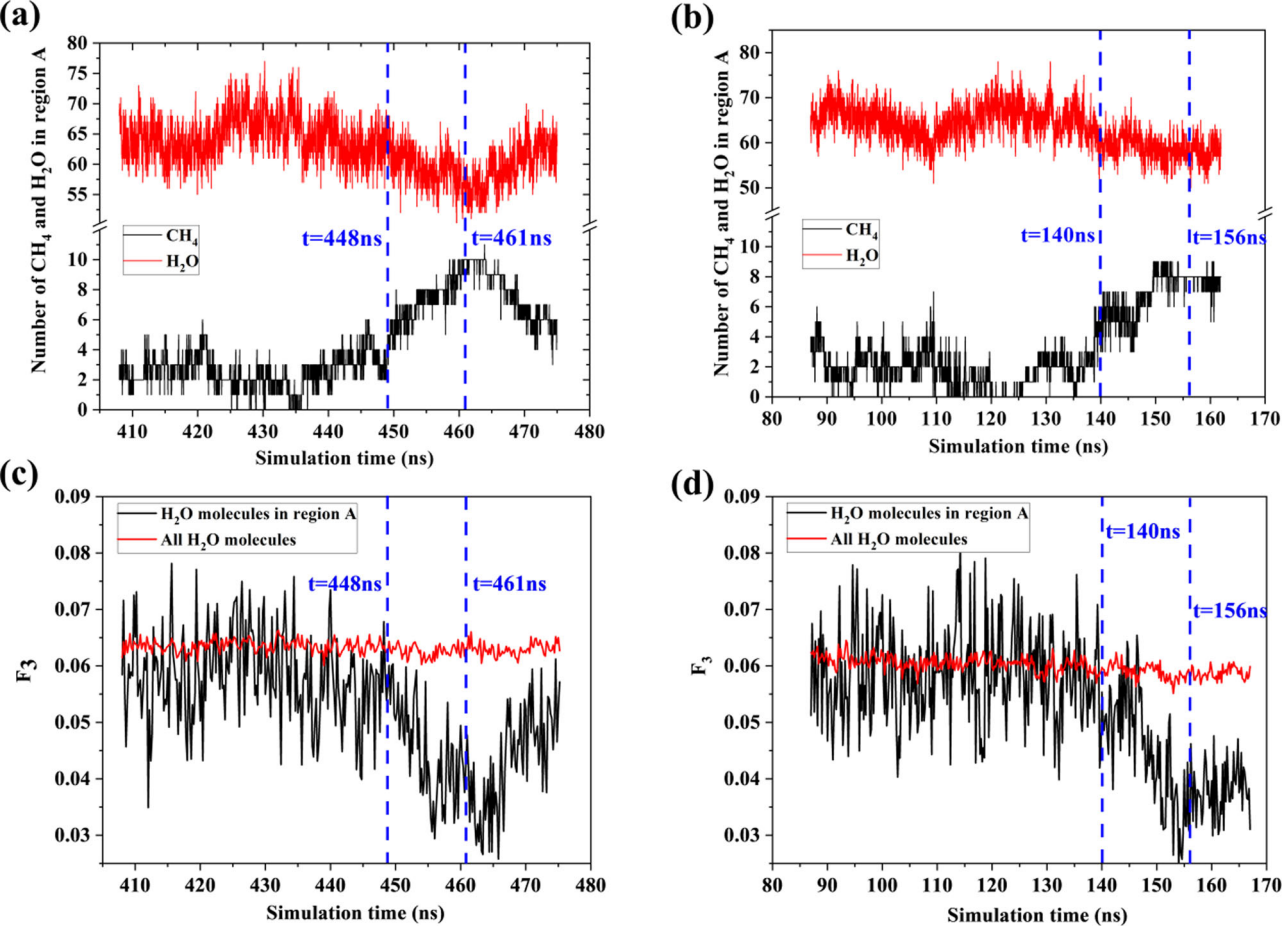
Methane distribution around central methane in boxes A and C. **a**, **b** The numbers of methane and water molecules in region A as a function of simulation time for boxes A and C. **c**, **d** Variation of *F*_3_ order parameter for boxes A and C. Both results for all water and water in region A are shown. **e** Change of numbers of methane and water molecules in the L1–L5 layers for box C.

**Fig. 5:**
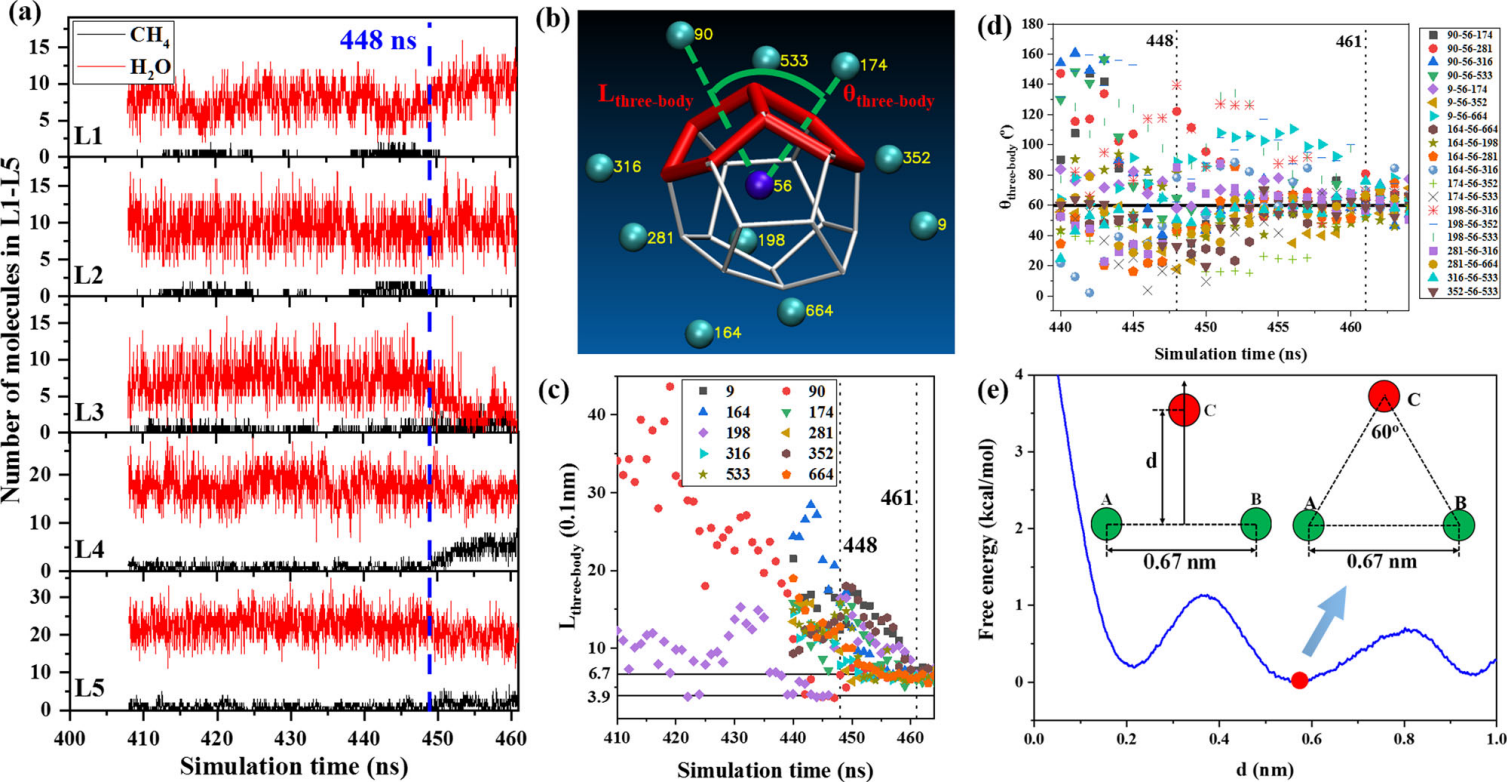
Gas aggregation characteristics around the central methane during hydrate nucleation (box A). **a** Change of numbers of methane and water molecules in the L1–L5 layers. The layers are distributed by the distance to central methane. L1 ≤ 0.4 nm, 0.4 nm < L2 ≤ 0.5 nm, 0.5 nm < L3 ≤ 0.6 nm, 0.6 nm < L4 ≤ 0.7 nm, 0.7 nm < L5 ≤ 0.8 nm. **b** A typical formed methane hydrate 5^12^ cage and the neighbor methane in region A. Balls represent different methane molecules (the central methane: blue; others: cyan) and their ids are recorded in yellow. A five-membered ring as well as its neighbor with one common ring are marked in red. **c** Change of *L*_three-body_ during hydrate nucleation. Two methane with id 90 and 198 are shown from 410 ns and the rest are shown from 440 ns for clarity. **d** The angle *θ*_three-body_ varying with time during hydrate nucleation. **e** Free energy of the three-body interactions for methane in water at 50 MPa and 250 K. Methane A, B are frozen and methane C locates at the perpendicular bisector of methane A and B. *d* is a reaction coordinate which is the vertical distance of methane C with A and B. The red ball indicates the minimum of free energy curve.

The gas aggregation trajectory around the central methane during hydrate nucleation has been analyzed. The central methane is the one which locates in the center of cage when the first hydrate cage is formed. The first complete hydrate cage was identified by analyzing molecular dynamics simulation trajectories. We track the migration history of the central methane before the hydrate cage is formed. The region within 0.8 nm near the central methane is defined as region A. As shown in Fig. 4a, b, at first, the numbers of methane and water molecules fluctuate; then methane molecules gradually approach to the central methane and at the same time, water molecules move away from the central methane due to adsorption layer compression^24^. Figure 4c, d illustrate that when gas aggregates towards the central methane, the *F*_3_ order parameter of water molecules in region A decreases significantly which directly indicates that the structural order of water molecules is inseparable from the proximity of methane molecules. To further investigate how methane molecules gather, region A is divided into several layers and Fig. 5a illustrates the numbers of methane and water molecules in these layers. Before nucleation, there is one or two methane within 0.4 nm of the central methane. After 448 ns (the moment when *F*_3_ of water in region A starts to decrease, Fig. 4c), the methane in L1 and L2 layers gradually move out, and at the meantime, the number of methane in L4 layer (0.6–0.7 nm) continues to increase. During nucleation, the numbers of water in L2 and L4 layers fluctuate but no significant variation has been found. More water molecules move into L1 layer while the number of water molecules in L3 is significantly reduced. At 461 ns, the numbers of water in L1 and L2 are ~12 and ~8, respectively, which agrees well with the structure of sI hydrate cage^35^. The numbers of water and methane in L5 slightly change but it is not significant. The maximum number of methane molecules reduced in L1 and L2 is 4 while the maximum number of methane molecules increased in L3 and L4 is 8, indicating that the increased methane molecules in L3 and L4 not only come from L1 and L2, but also from outer space. Similar characteristics have been revealed and the results for box C are illustrated in Supplementary Fig. 4.

Gas aggregation pattern of the first hydrate cage during nucleation has been further investigated. In all simulation runs, 5^12^ cages are always formed prior to 5^12^6^2^ and 5^12^6^4^, so only the aggregation around the 5^12^ hydrate cage is analyzed. The neighbor CH_4_ molecules which are located within 0.8 nm from the central methane when the hydrate cage is formed, were marked and the result for box A is shown in Fig. 5b. Ten methane were found around the hydrate cage. Each of them is located at the opposite side of one five-membered ring corresponding to the central methane and they will be referred to as “directional methane” of the five-membered rings. A three-body pattern is formed by the central methane and the two directional methane of two neighbor five-membered rings. The aggregation geometry of these three-body methane was analyzed using *L*_three-body_ and *θ*_three-body_ where *L*_three-body_ denotes the distance between the central methane and one directional methane and *θ*_three-body_ represents the angle formed by the three methane molecules (centered at the central methane). Figure 5c displays *L*_three-body_ of the 10 directional methane as a function of time. From 448 ns, the directional methane molecules aggregate towards the central methane and the final distances are ~6.7 Å which equals the peak distance of radial distribution functions between methane and methane molecules in pure methane hydrate (Supplementary Fig. 5) and the solvent-separated methane–methane pair in water^36,37^. Two methane molecules (with id 198 and 90) have been close to the central methane at ~3.9 Å (the distance of a contact methane–methane pair in water^37^) for several ns before nucleation. Although the nature is unclear, it is well known that the hydrophobic interaction of methane in gas hydrate is governed by solvent separated pairs^38^. They tend to be solvated by water upon nucleation from contact to solvent separation interaction with the central methane. Figure 5d shows the variation of *θ*_three-body_ with time. Although the angles frequently fluctuate, they favor being ~60° when the directional methane molecules aggregate towards the central methane. For a 5^12^ cage in the crystalline sI methane hydrate which has the full 12 directional methane, the corresponding *L*_three-body_ ranges from 6.6 to 7.0 Å with an average 6.7 (0.2) Å and the corresponding *θ*_three-body_ is 49.8°–67.9° with an average 63.7 (5.4)°. The difference of *θ*_three-body_ in the nucleation process and the crystalline state is not surprising. During nucleation the directional methane molecules have not been captured by hydrate cages and other cages will continue to form which causes *L*_three-body_ and *θ*_three-body_ to slightly fluctuate. Similar evolutionary characteristics of *L*_three-body_ and *θ*_three-body_ for box C have been found and the results are illustrated in Supplementary Fig. 6. By fixing the separation of two methane at 6.7 Å, the free energy of the three-body interaction of methane molecules was calculated as illustrated in Fig. 5e (refer to the “Methods” section for the calculation of free energy). It has been shown that for three-body interaction the optimally packed cluster can only form when the third methane is placed symmetry relative to the methane dimer^37^. As a result, we select the distance between the center of two frozen methane and the third methane as a reaction coordinate. The free energy minimum locates at *d* = 0.58 nm showing that the three methane molecules favor forming a regular triangle, which agrees well with the gas aggregate pattern in Fig. 5c, d. This implies that during nucleation, methane molecules aggregate with a three-body solvent separated pattern. The three methane molecules which aggregate with this pattern are referred to as a “three-body aggregate”.

**Fig. 6:**
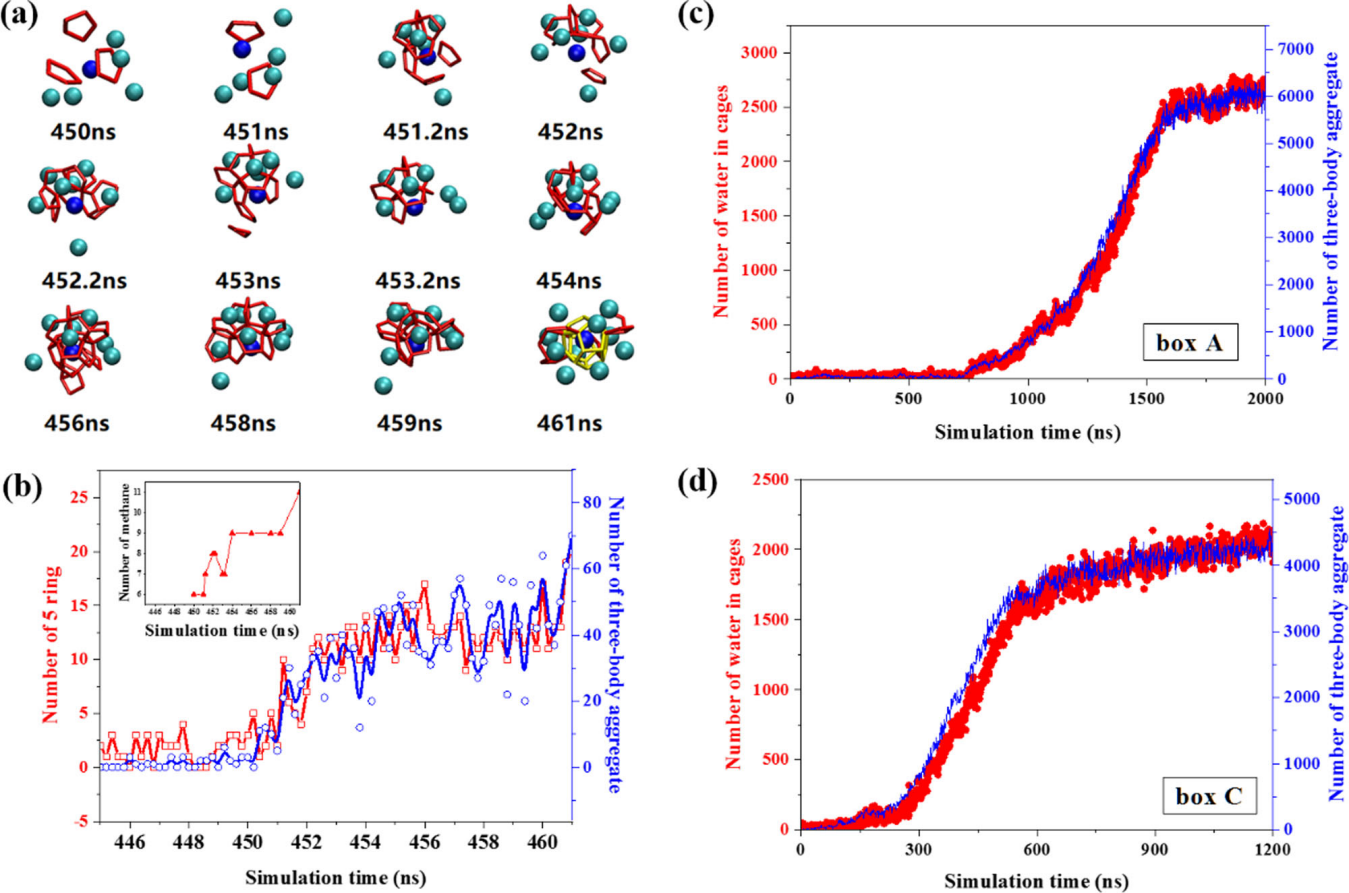
Snapshots of gas aggregation and evolution of the number of three-body aggregates. **a** Gas aggregation process in region A which is within 0.8 nm of the central methane. The central methane is represented by the blue ball. The red lines indicate the five-membered rings and the yellow lines represent a complete 5^12^ cage. **b** The number of five-membered rings and the three-body aggregates structure in region A during nucleation. The number of methane molecules in the same region was drawn in the inner picture. **c**, **d** Number of water in cages as well as the number of three-body aggregates during hydrate nucleation and growth for boxes A and C, respectively.

Figure 6a illustrates the gas aggregation process in region A. The formation of cages is significantly related to the proximity of methane. Figure 6b shows the numbers of methane, five-membered rings and three-body aggregates in region A. As methane aggregates, the number of five-membered rings gradually increases. However, the number of five-membered rings is not directly proportional to the degree of methane accumulation. From 454 to 459 ns, there are nine methane molecules around the central methane; however, the number of five-membered rings changes from 9 to 17. The variation of the number of five-membered rings is caused by the changing of gas aggregation pattern, as shown in Fig. 5c, d. The number of five-membered rings is highly correlated with the number of three-body aggregates. As more methane molecules are involved in the three-body aggregates, the number of five-membered rings increases accordingly. Figure 6c, d demonstrate the number of water in cages and the three-body aggregate number as a function of simulation time (refer to the “Methods” section for the identification of three-body aggregates). It is found that the evolution of the number of water in cages is consistent with that of three-body aggregate. The good consistency is also obtained for six independent runs of box A as summarized in Supplementary Fig. 7. It shows that the growth of hydrate is inextricably linked to the three-body aggregates. It should be noted that the aggregation characteristics of guest molecules during hydrate nucleation were found in six independent simulations for each simulation box except for the typical time related with different induction times (Supplementary Table 2).

### Effects of three-body aggregates on gas hydrate nucleation and growth

**Fig. 7:**
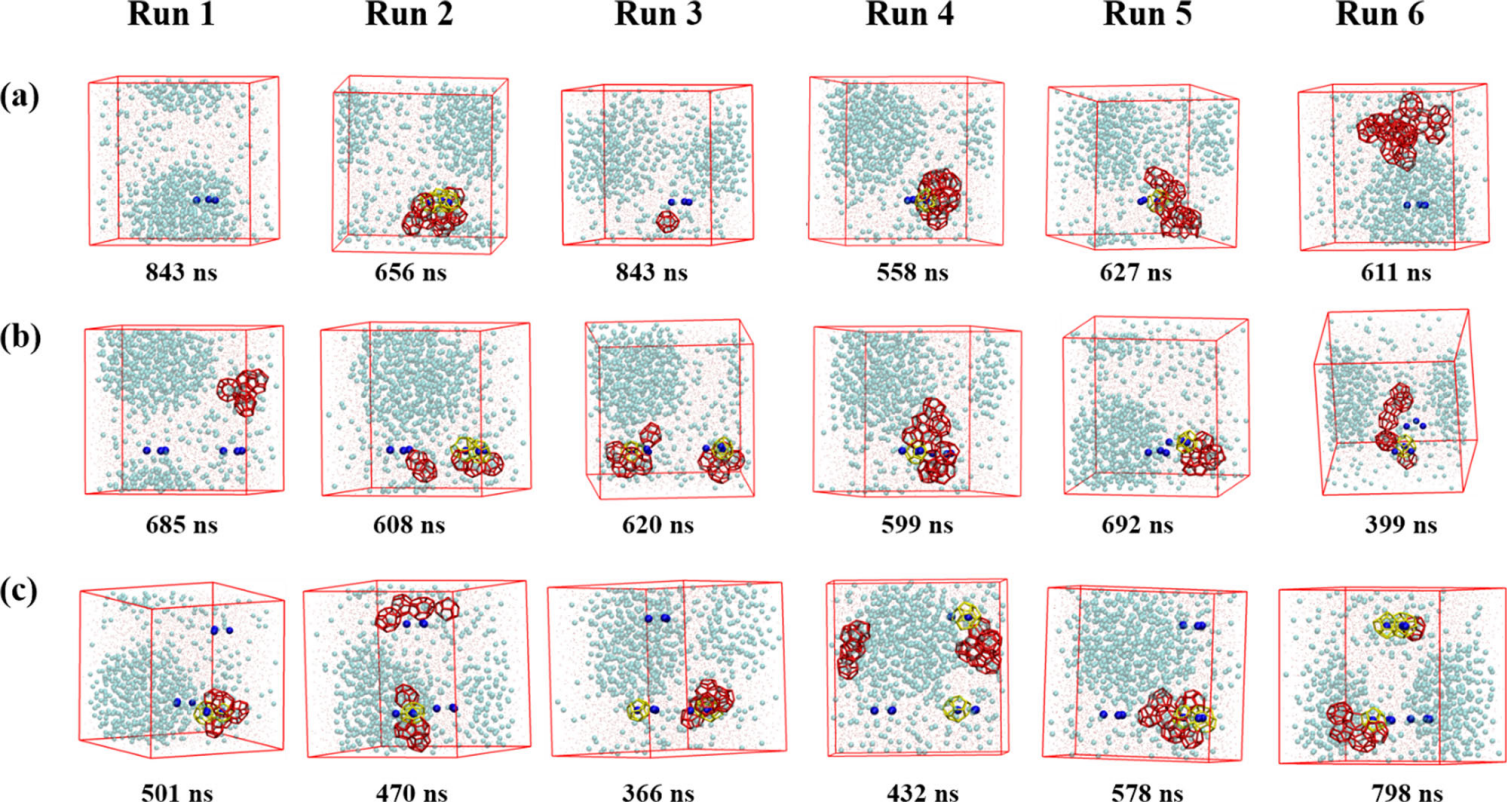
Effects of three-body aggregates on hydrate nucleation sites. Different number of three-body aggregates were added into the initial configuration and the corresponding numbers of methane molecules were removed to ensure the consistency of the number of molecules in the system. The methane molecules in the three-body aggregates were fixed during simulation to keep their aggregation patterns. For each system, six independent runs were performed. **a**–**c** display the results for systems with one, two and three three-body aggregates, respectively. The snapshots when the number of hydrate cages becomes larger than 10 are present. If the number of hydrate cages is always smaller than 10, the snapshot at final status is provided. The blue balls represent methane molecules in the three-body aggregates. The simulation boxes are rotated for clarity. The hydrate cages which central methane is a part of the three-body aggregate is marked in yellow; otherwise, the cages are drawn in red.

To reveal the effect of three-body aggregate on hydrate nucleation and growth, systems with different numbers of three-body aggregates were constructed. Figure 7 displays the effects of three-body aggregates on hydrate nucleation sites. Three-body aggregation of methane molecules is a key step for hydrate nucleation; however, it is not the sufficient condition. More methane molecules are required to gather around to form a hydrate cage. It is still possible for those methane molecules at other locations to form three-body aggregates by themselves. If only one three-body aggregate is initially provided, hydrate nucleates at the three-body aggregate in 3/6 (runs 2, 4, 5) of the independent runs. In the case of two three-body aggregates, hydrate nucleation was triggered by the three-body aggregates for 5/6 (runs 2–6) of the performed runs. In all the six independent runs, hydrates were found to nucleate at the three-body aggregate if there are initially three three-body aggregates in the system. It takes a long time for gas molecules to spontaneously accumulate into “three-body aggregates” as shown in Fig. 6c, d. By placing a three-body aggregate, the possibility of other methane molecules to aggregate with a three-body solvent separated pattern was enhanced. The possibility is further strengthened by enlarging the number of initial three-body aggregates. Two of the aggregates both trigger hydrate nucleation in run 3 for the system with two aggregates and runs 3, 4 and 6 for the system with three aggregates. However, nucleation at three aggregates simultaneously was not found in these systems.

**Fig. 8:**
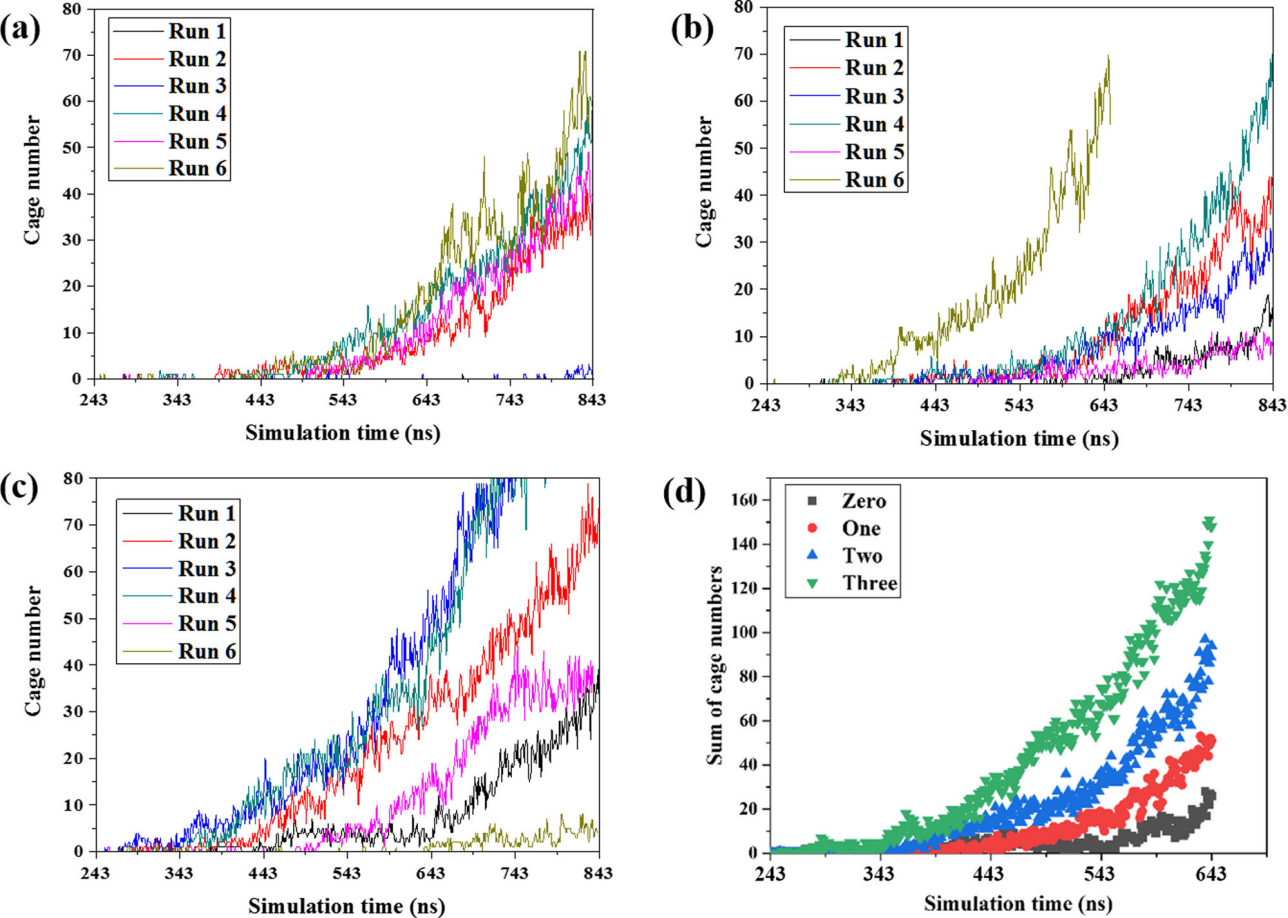
Acceleration of three-body aggregates on hydrate nucleation and growth. The total cage numbers were calculated and the results for one, two, and three three-body aggregates systems are illustrated in (**a**–**c**), respectively. **d** The cage numbers for the six independent runs were summed and compared for systems with zero, one, two, and three aggregates.

Figure 8a–c demonstrates the cage numbers as a function of simulation time for different systems with one, two, and three three-body aggregates. Due to the spontaneous nature of hydrate nucleation, the induction time and growth speed show deviations for different runs even the initial systems are the same^24^. However, the acceleration of three-body aggregates on hydrate nucleation and growth is still clear. The sum of cage numbers for six runs is illustrated in Fig. 8d. It is rather obvious that three-body aggregates enhance hydrate nucleation and growth. As the number of three-body aggregates increases in the initial system, the hydrate nucleation induction time becomes shorter and the hydrate grows at a faster rate. At 443 ns, the average numbers of hydrate cages for systems with initially two and three three-body aggregates are ~4.3 and ~10.3 times of that for the system with only one three-body aggregate, respectively. The ratios decrease to ~2.6 and ~5.4 at 543 ns. It takes ~633 ns for the system with one three-body aggregate to form 50 cages. For the systems with two and three three-body aggregates, the period has been shortened by ~16% and ~38%, respectively. For systems containing zero, one, two and three three-body aggregates, the nucleation rates of the hydrate in the four systems are 7.91 × 10^24^, 7.91 × 10^24^, 9.57 × 10^24^, and 1.18 × 10^25^ nuclei cm^−3^ s^−1^, respectively, which agree well with the predicted data by Walsh et al. ^32^. The nucleation rate is nearly affected by placing only one three-body aggregate. However, it seems that by placing two and three three-body aggregates, the nucleation rate increases by 21% and 49%, respectively, compared with the system without any three-body aggregate.

## Conclusion

In summary, we constructed different methane–water boxes to investigate the effects of nanobubble on hydrate nucleation and growth. The evolution behavior was controlled by the initial gas–water ratio. At larger gas–water ratios, a plateau was found for curves of potential energy, methane concentration, and water number in cages, indicating a relative equilibrium between gas bubble and water solution in the induction period. Gas hydrate nucleates at several nm away from the gas–water interface at a molecular level as the methane concentration is low and *F*_3_ order parameter is high at the interface.

The gas aggregation trajectory was tracked and evolution of methane in different layers around the central methane show distinct features. During nucleation, methane in layers (≤0.5 nm) gradually move out, while the number of methane in an outer layer (0.6–0.7 nm) continues to increase. Gas aggregation pattern analysis demonstrates that a regular triangle pattern is formed by the central methane and the two directional methane of two neighbor five-membered rings. The length of the triangle is ~6.7 Å which equals the peak distance of C–C radial distribution functions in methane hydrate and the solvent separated methane–methane pair in water. The regular triangle pattern is confirmed as the free energy minimum for three body interaction of methane molecules in water when two of them are solvent separated. The variation of the three-body aggregates of guest molecules is well consistent with that of five-membered rings and number of water in cages. By providing different numbers of three-body aggregates, the nucleation sites and hydrate growth rate have been significantly regulated. If one, two, and three three-body aggregates are initially provided, hydrate nucleation triggers at these three-body aggregates in 3/6, 5/6, and 6/6 of the independent runs, and in some cases, more than one aggregates trigger hydrate nucleation. As the number of three-body aggregates increases in the initial system, the hydrate nucleation induction time becomes shorter and the hydrate grows at a faster rate. The results demonstrate three-body aggregation of guest molecules as a key step in gas hydrate nucleation and growth. However, what causes the methane molecules aggregate into the found pattern is unclear. We did search the trajectory of these methane molecules, however, the structure of methane–water solutions is rather complicated under high pressure and low temperature conditions. As shown by Guo et al. ^39^, there are thousands of cage types occurring in methane aqueous solution and in hydrate nucleation processes. Even we identify all the cage types, it is hard to distinguish what “cages” are the main cause of the three-body aggregate. As the cages and water molecules around the methane molecules vary frequently with time and the existence of cage affects the formation of other cages. Among the theories related with methane–water interaction, the cage adsorption hypothesis is most probably the reason to cause three-body aggregate. Future studies are required to investigate what cages and how to adsorb methane molecules to form the three-body aggregate and thus to control the gas hydrate nucleation and growth.

## Methods

### MD simulation

A system shown in Supplementary Fig. 1a was built. All molecular dynamics simulations were performed by nanoscale molecular dynamics (NAMD)^40^. The CHARMM format potential energy function was used to calculate the total potential energy of the system. For CH_4_, we adopt the united-atom model TraPPE^41^, and for H_2_O, we adopted the four-point rigid model TIP4P/ice^42^. The detailed force field parameters are listed in Supplementary Table 3. TraPPE–TIP4P/ice model combination was proved to predict the hydrate phase diagram^43^ successfully and has been applied by several authors^15,44,45^. In all simulations, the periodic boundary conditions were applied in the three directions of *x*, *y,* and *z* to eliminate boundary effects. The initial velocities of particles satisfied the Maxwell–Boltzmann distribution. The cut-off radius is set to be 1.2 nm and the time step was set to be 2.0 fs. The particle mesh Ewald^46^ (PME) method was used to calculate the Coulombic interactions for full system and the desired relation error in force was set to be 10^−6^. The non-iterative SETTLE algorithm is used to keep the rigidity of water^47^. The pressure of the system was maintained using the Langevin position^48^ method with a Langevin piston period of 100 time steps and a Langevin piston decay of 50 time steps. The temperature of the system was controlled by Langevin dynamic^49^ method with a damping coefficient of 5 ps. The system runs thousands of nanoseconds at 50 MPa and 250 K to output data. The in-house codes written by our team were used to analyze the data and VMD software was used to visualize the simulation results^50^. Hydrate cages were identified according to the computational code of Sum^51^. It should be noted that a thermostat has been applied which may affect the transport coefficients in liquids, however, the gas aggregation patterns found in this manuscript are not influenced.

### Identification of three-body aggregates

The three-body aggregate is composed of three methane molecules in the liquid phases, and these three methane have a certain positional relationship. With methane 1 as the center, methane 2 and methane 3 are at a distance of 0.55–0.75 nm from methane 1, and the angle between them is 50°–70° (with methane 1 as the vertex).

### Identification of CH_4_ phase

The number of solvent molecules around methane in gas and liquid is different. By calculating the RDF of CH_4_–H_2_O in methane–water dilute solution, it was found the number of water molecules in the first solvation shell of methane is about 20 within 5.5 Å. Therefore, a methane molecule was defined as gas phase if the number of water molecules in the range of 5.5 Å of it is <11. A methane molecule with more than 10 water-like water molecules in the first hydration shell was considered to be in the solution phase.

### Identification of H_2_O phase

The phase of water molecules can be identified by calculating the angular order parameters of water molecules and counting the number of water rings that water molecules participate in. AOP is an angular order parameter consisting of three water molecule configurations that describe the degree of deviation of a tetrahedron formed by a central oxygen atom and other oxygen atoms from the regular tetrahedron, which can be defined as^52^

$${{{{{\rm{AOP}}}}}}=\mathop{\sum }\limits_{{j}=1}^{{{n}}_{{i}}-1}\mathop{\sum }\limits_{{k}={j}+1}^{{{n}}_{{i}}}{\left({|}{{\cos }}\,{{\theta }}_{{jik}}{{{{{\rm{|}}}}}}{{\cos }}\,{{\theta }}_{{jik}}+{\left({{\cos }}\,{109.47}^{^\circ }\right)}^{2}\right)}^{2}$$

where ${{\theta }}_{{jik}}$ donates the angle between the oxygen atoms of water molecule *j*, *i* and *k*; ${109.47}^{^\circ }$ indicates the angle between the center of the regular tetrahedron and the vertices. The cut-off distance between the oxygen atom and its nearest neighbors is 3.5 Å, corresponding to the first minimum in the water oxygen–oxygen radial distribution function of the hydrate phase. If a water molecule with AOP < 0.4 and participates in 4 or 5 or 6 five-membered rings, the water molecule was defined as hydrate phase. After distinguishing water molecules into hydrate phase or liquid phase using the above method, it is necessary to perform a second identification. If a water molecule in hydrate has three or more adjacent water molecules in liquid (the adjacent distance is 3.5 Å), the water molecule in hydrate will be revised as liquid water; if a water molecule in liquid has three or more adjacent water molecules in hydrate (the adjacent distance is 3.5 Å), the water molecule in the liquid will be revised as hydrate water.

### *F*_3_ Order parameter

The *F*_3_ order parameter is also an angular order parameter to describe the degree of deviation of a tetrahedron formed by a central oxygen atom and other oxygen atoms from the regular tetrahedron. The *F*_3_ order parameter has been commonly used to characterize hydrate structures in the previous research. Unlike AOP, the calculation of *F*_3_ order parameter involves the averages of values, which can be defined as^13,27,53^

$${{F}}_{3}=\frac{{{{{{\rm{AOP}}}}}}}{{{n}}_{{i}}\left({{n}}_{{i}}-1\right)/2}$$

where ${{n}}_{{i}}$ represents the nearest neighbors of water molecule *i*. For water molecules in liquid, *F*_3_ = 0.09; for water molecules in hydrate, *F*_3_ = 0.01.

### Free energy calculation

The free energy of the three-body interactions for methane was calculated by the extended-system adaptive biasing forces (eABF)^54^, which is a variant of the adaptive biasing forces (ABF) method^55^, which can overcome the limitations of the traditional ABF method and improve the convergence speed of the simulation. As shown in Fig. 5e, in the calculation process, methane A and B are fixed, and methane C is located at the perpendicular bisector of methane A and B. The used colvar (collective variables) component is distance. The reaction pathway *d* is defined as the distance from methane C to the center of methane A and B. The lower boundary of *d* is 0, the upper boundary of *d* is 10, and the calculated width is 0.01. The units of lower boundary, upper boundary and width were Å. The lower and upper wall constant are set to be 100.0, and the unit is kcal mol^−1^. The movement of methane C in the *y* and *z* directions is restricted.

## Supplementary information

Supplementary information

## Data Availability

The initial and final configurations of molecular dynamics trajectories were provided in ScienceDB with the access link as: https://www.scidb.cn/s/6R32Uv. Other data that support the findings of this study are available from the corresponding author upon reasonable request.

## Code availability

The code for analyzing three-body aggregates that support the findings of this study are available from the corresponding author upon reasonable request.
