# Supplementary material for: Three-body aggregation of guest molecules as a key step in methane hydrate nucleation and growth
Source: Commun Chem. 2022 Mar 14;5:33. doi: 10.1038/s42004-022-00652-0 (PMC9814777; doi:10.1038/s42004-022-00652-0)
Supplement: Supplementary file 1 — Supplementary information [file 42004_2022_652_MOESM1_ESM.pdf]

Supporting Information for

"Three-body aggregation of guest molecules as a key step in methane hydrate nucleation and growth"

Wenfeng Hu[1], Cong Chen[1,2,*], Jingyue Sun[1], Ning Zhang[3], Jiafei Zhao[1,2], Yu Liu[1,2], Zheng Ling[1,2], Weizhong Li[1,2], Weiguo Liu[1,2], Yongchen Song[1,2,*]

[1]School of Energy and Power Engineering, Dalian University of Technology, Dalian 116024, P. R. China

[2]Key Laboratory of Ocean Energy Utilization and Energy Conservation of Ministry of Education, Dalian 116024, P. R. China

[3]School of Petroleum and Chemical Engineering, Dalian University of Technology, Panjin 124221, P. R. China

*Corresponding author. Email: congchen@dlut.edu.cn

(a)

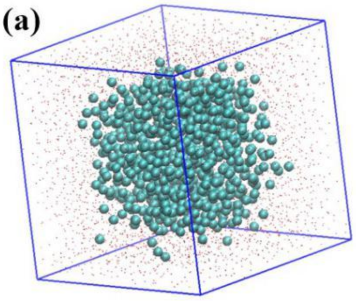

(b)

Box	$N(H_2O)$	$N(CH_4)$	Bubble size (nm)	CH_4 molar fraction
A	3881	675	2.125	0.148
B	4250	512	1.936	0.108
C	4820	456	1.821	0.086

Supplementary Figure 1. A snapshot and composition of initial simulation boxes. **(a)** Configuration of the initial simulation box. A methane nanobubble was placed in the center of a cubic water box with a length of 5.4 nm. Three simulation boxes with different size of nanobubbles were built for comparison. **(b)** Parameters of different CH_4-water boxes. As for box A, the molar concentration of methane in water is 0.148, which corresponds to the methane concentration in a full-occupied sI methane hydrate.

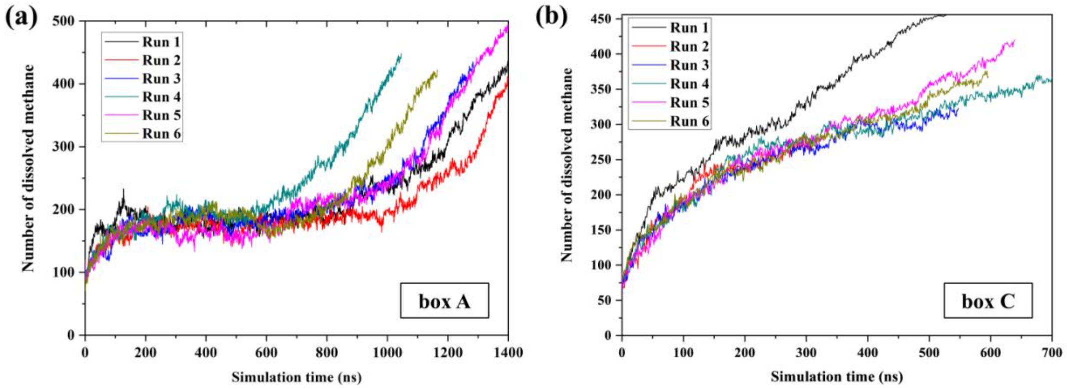

Supplementary Figure 2. The evolution of liquid methane in the process of hydrate nucleation for different boxes. **(a)** The number of methane molecules dissolved in liquid water as a function of simulation time for box A. Six independent runs were performed. **(b)** The number of methane molecules dissolved in liquid water as a function of simulation time for box C.

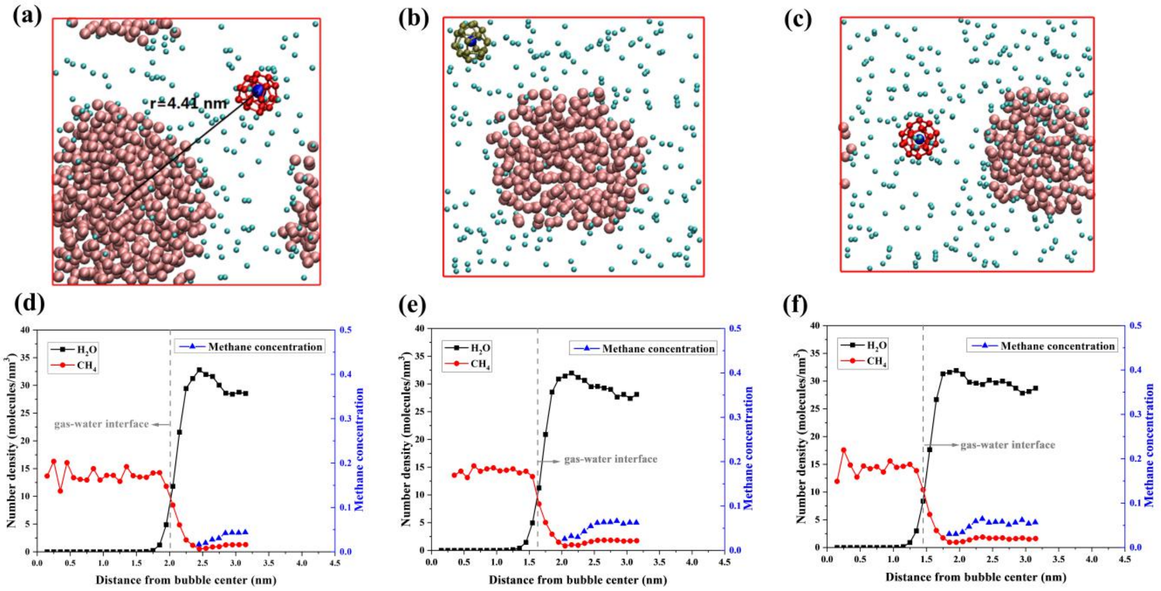

Supplementary Figure 3. **The first complete hydrate cage, water and methane density, methane concentration around the bubble. (a-c)** The snapshots of box A, B and C when the first complete cage formed. Red and tan: Oxygen atoms in cage; Blue: Methane in cage; Pink: Methane in bubble; Cygan: Methane in bulk water. **(d-f)** The distribution of water and methane number density as well as methane concentration as a function of distance from bubble center for box A, B and C, respectively. The data are collected at 440-460 ns, 172-192 ns and 135-155 ns for boxes A, B and C, respectively.

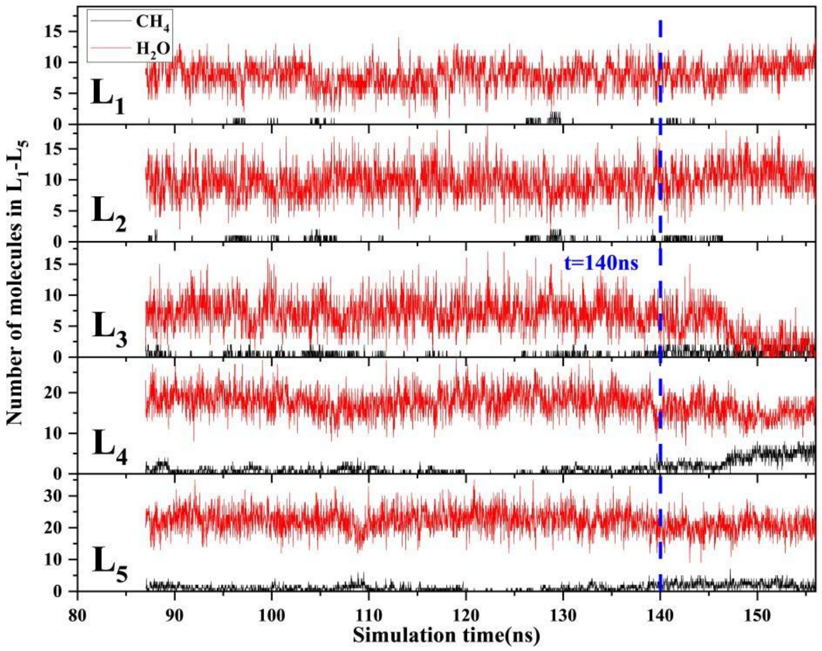

Supplementary Figure 4. Change of numbers of methane and water molecules in the L1-L5 layers of box C.

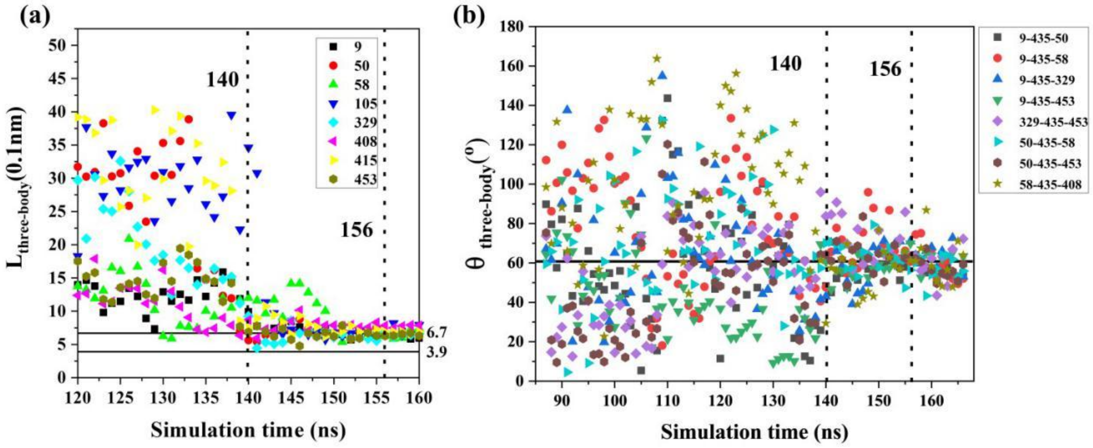

Supplementary Figure 5. The change of $L_{three-body}$ and $\theta_{three-body}$ during the formation of 5^{12} cage in box C. (a) Change of $L_{three-body}$ during hydrate nucleation. The hydrate cage was formed at 156 ns. From 140ns, the directional methane molecules aggregate towards the central methane and the final distances are ~6.7 Å. **(b)** The angle $\theta_{three-body}$ varying with time during hydrate nucleation. From 140ns, the angles $\theta_{three-body}$ favor being ~60 ° when the directional methane molecules aggregate towards the central methane. The change characteristics of $L_{three-body}$ and $\theta_{three-body}$ are similiar to box A.

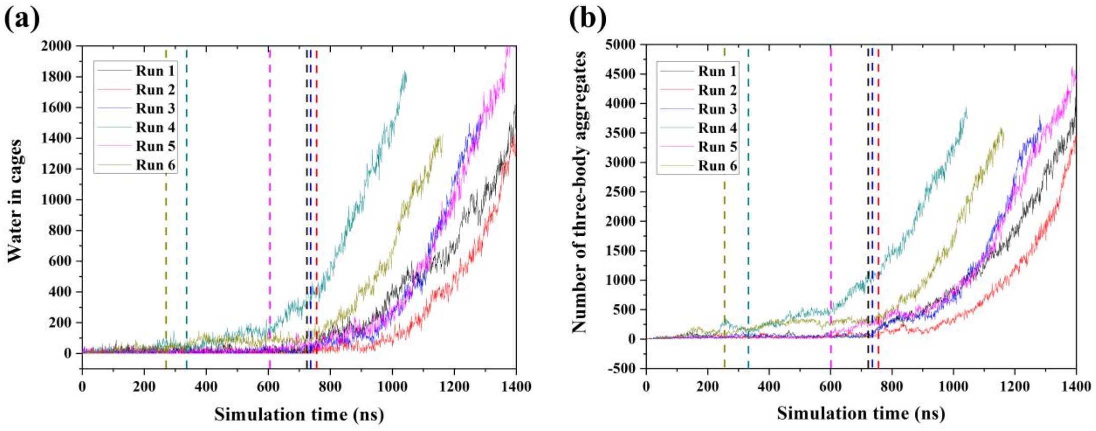

Supplementary Figure 6. High correlation between the growth rate of hydrate and the three-body aggregate structure. (a) The number of water molecules in cages changes with time in six independent runs. **(b)** The number of three-body aggregates structure changes with time in six independent runs. The dotted line indicates the start time of hydrate growth in each independent run.

Supplementary Table 1. The rate of hydrate growth and the rate of concentration increase in different stages. w represents the increase of the number of water in cages per ns and c represents the increase of the liquid methane mole fraction per ns.

Box	Growth phase					
	Stage 1		Stage 2		Stage 3	
	w	c(10^{-5})	w	c(10^{-5})	w	c(10^{-5})
A	0.94	6.47	3.33	49.87	0.24	9.66
B	1.08	9.57	3.44	28.06	0.68	5.85
C	0.71	5.77	2.80	20.18	0.39	0.82

Supplementary Table 2. The induction time of box A and C in 6 simulations.

	Induction time						
	Run 1	Run 2	Run 3	Run 4	Run 5	Run 6	Average
box A	764ns	980ns	974ns	523ns	894ns	579ns	785.7ns
box C	218ns	290ns	431ns	555ns	340ns	389ns	370.5ns

Supplementary Table 3. Force field parameters of CH_4 and H_2O.

Model		q/e	$\varepsilon / kcal \cdot mol^{-1}$	$\sigma / \mathring{A}$	$K_r / kcal \cdot mol^{-2} \cdot \mathring{A}^{-2}$	$K_\theta / kcal \cdot mol^{-2} \cdot rad^{-2}$	$l / \mathring{A}$	$angle/\circ$
TraPPE(CH_4)		0.000	0.294076	3.73				
TIP4P/ice(H_2O)	H	0.5897	0.00	0.00				
	O	-1.1794	0.210821	3.1668				
	O-M						0.1577	
	O-H				450	33	0.9572	
	H-O-H							104.52
